# Poly[hemi-μ-aqua-[μ-2-(2-bromobenzenesulfonamido)benzoato]sodium(I)]

**DOI:** 10.1107/S1600536809028505

**Published:** 2009-07-25

**Authors:** Islam Ullah Khan, Muhammad Nadeem Arshad, Mehmet Akkurt, Ghulam Mustafa, Muhammad Shafiq

**Affiliations:** a Department of Chemistry, Government College University, Lahore, Pakistan; b Department of Physics, Faculty of Arts and Sciences, Erciyes University, 38039 Kayseri, Turkey

## Abstract

The asymmetric unit of the title compound, [Na(C_13_H_9_BrNO_4_S)(H_2_O)_0.5_]_*n*_, contains two Na^+^ cations, two substituted benzoate anions and one water molecule of crystallization. The Na^+^ cations are coordinated in an octa­hedral geometry by two carboxyl­ate O atoms, two sulfonyl O atoms and two water O atoms. The latter two ligands occupy *trans* positions. The polymeric network structure of the title complex is characterized by a layered assembly parallel to (001) and is further consolidated by N—H⋯O, O—H⋯O and C—H⋯O hydrogen-bonding inter­actions.

## Related literature

For the physical properties of metal complexes of anthranilic acid derivatives, see: Chacko & Parameswaran (1984 ▶). For 2-(4-bromo­benzene­sulfonamido) benzoic acid, see: Arshad *et al.* (2009 ▶).
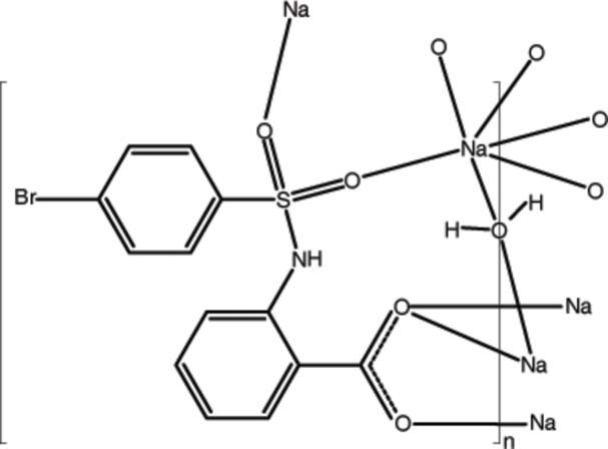


## Experimental

### 

#### Crystal data

[Na(C_13_H_9_BrNO_4_S)(H_2_O)_0.5_]
*M* *_r_* = 387.18
Triclinic, 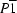

*a* = 9.1683 (6) Å
*b* = 9.2722 (5) Å
*c* = 18.4183 (12) Å
α = 97.717 (2)°
β = 101.837 (2)°
γ = 101.467 (2)°
*V* = 1476.64 (16) Å^3^
*Z* = 4
Mo *K*α radiation
μ = 2.97 mm^−1^
*T* = 296 K
0.21 × 0.12 × 0.10 mm

#### Data collection

Bruker Kappa APEXII CCD area-detector diffractometer
Absorption correction: multi-scan (*SADABS*; Bruker, 2007 ▶) *T*_min_ = 0.574, *T*_max_ = 0.755
28784 measured reflections
6503 independent reflections
3926 reflections with *I* > 2σ(*I*)
*R*_int_ = 0.045

#### Refinement

*R*[*F* ^2^ > 2σ(*F* ^2^)] = 0.049
*wR*(*F* ^2^) = 0.125
*S* = 1.01
6503 reflections
394 parameters
3 restraints
H atoms treated by a mixture of independent and constrained refinement
Δρ_max_ = 0.79 e Å^−3^
Δρ_min_ = −0.78 e Å^−3^

### 

Data collection: *APEX2* (Bruker, 2007 ▶); cell refinement: *SAINT* (Bruker, 2007 ▶); data reduction: *SAINT*; program(s) used to solve structure: *SIR97* (Altomare *et al.*, 1999 ▶); program(s) used to refine structure: *SHELXL97* (Sheldrick, 2008 ▶); molecular graphics: *ORTEP-3 for Windows* (Farrugia, 1997 ▶); software used to prepare material for publication: *WinGX* (Farrugia, 1999 ▶) and *PLATON* (Spek, 2009 ▶).

## Supplementary Material

Crystal structure: contains datablocks global, I. DOI: 10.1107/S1600536809028505/fj2240sup1.cif

Structure factors: contains datablocks I. DOI: 10.1107/S1600536809028505/fj2240Isup2.hkl

Additional supplementary materials:  crystallographic information; 3D view; checkCIF report

## Figures and Tables

**Table 1:** Hydrogen-bond geometry (Å, °)

*D*—H⋯*A*	*D*—H	H⋯*A*	*D*⋯*A*	*D*—H⋯*A*
N1—H1⋯O3	0.86	1.88	2.588 (3)	138
O9—H*W*1⋯O8	0.849 (19)	1.97 (3)	2.768 (3)	156 (6)
N2—H2⋯O7	0.86	1.90	2.595 (4)	137
O9—H*W*2⋯O4^i^	0.86 (4)	1.91 (4)	2.730 (3)	160 (6)
C2—H2*A*⋯O2	0.93	2.52	2.886 (4)	104
C8—H8⋯O2	0.93	2.55	3.117 (5)	120
C11—H11⋯O1^ii^	0.93	2.53	3.429 (4)	162
C11—H11⋯O4	0.93	2.41	2.732 (5)	100
C19—H19⋯O5	0.93	2.51	2.889 (5)	105
C22—H22⋯O8	0.93	2.41	2.736 (4)	101
C25—H25⋯O5	0.93	2.48	3.103 (5)	124

Symmetry codes: (i) 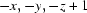; (ii) 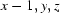.

**Fig. 1.**
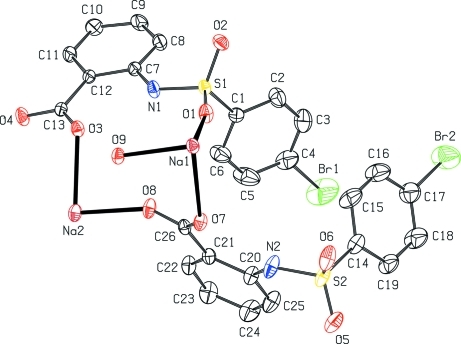
View of the title complex in the asymmetric unit, showing the atom labeling scheme and displacement ellipsoids drawn at the 30% probability level. H atoms have been omitted for clarity.

**Fig. 2.**
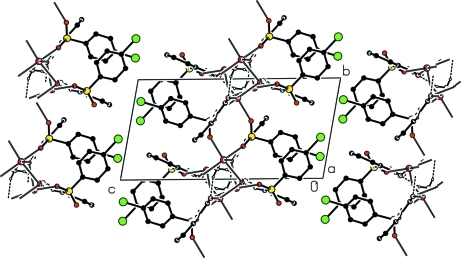
The crystal packing and hydrogen bonding (dashed lines) of the title complex, down the a-axis. H atoms not involved in the hydrogen bonding have been omitted for clarity.

## supplementary crystallographic information

### Comment

Metal complexes of anthranilic acid derivative specially the Schiff bases of the same precursor have been synthesized for studies their physical properties (Chacko & Parameswaran, 1984). The title complex is sodium salt of previously reported anthranillic acid derived sulfonamide 2-(4-bromobenzenesulfonamido) benzoic acid (Arshad *et al.*, 2009) asan intermediate for the synthesis of transition metal complexes.

In the title complex, (Fig. 1), the Na atoms occupy the voids in the network such that each exists in a six-coordinate geometry, by two carboxyl O atoms, two sulfonyl O atoms and two water O atoms. The forth coordination site of the tetragonal plane is occupied by two carboxyl O atoms, two sulfonyl O atoms; the apical sites are occupied by the water O atoms (Table 1). The network structure of the title complex is further consolidated by hydrogen-bonding interactions (Table2, Fig. 2).

### Experimental

2-(4-Bromobenzenesulfonamido)benzoic acid (1 g, 2.8 mmol) (Arshad *et al.*, 2009) was dissolved in distilled water and the pH was adjusted about 7–8. The white precipitate of title compound obtained was filtered and recrystalized in methanol.

### Refinement

The H atoms of the water molecule were found from a difference Fourier map and refined with distance restraints of O—H = 0.85 (1) Å and H···H = 1.39 (1) Å. The other H atoms were located geometrically and treated as riding, with C—H = 0.93–0.98 Å, N—H = 0.86 Å and O—H = 0.82 Å, with *U*_iso_(H) = 1.2*U*_eq_(C,*N*) and *U*_iso_(H) = 1.5*U*_eq_(O).

### Crystal data

**Table:**

[Na(C_13_H_9_BrNO_4_S)(H_2_O)_0.5_]	*Z* = 4
*M**_r_* = 387.18	*F*(000) = 772
Triclinic, *P*1	*D*_x_ = 1.742 Mg m^−^^3^
Hall symbol: -P 1	Mo *K*α radiation, λ = 0.71073 Å
*a* = 9.1683 (6) Å	Cell parameters from 6020 reflections
*b* = 9.2722 (5) Å	θ = 2.3–27.3°
*c* = 18.4183 (12) Å	µ = 2.97 mm^−^^1^
α = 97.717 (2)°	*T* = 296 K
β = 101.837 (2)°	Rod like, white
γ = 101.467 (2)°	0.21 × 0.12 × 0.10 mm
*V* = 1476.64 (16) Å^3^	

### Data collection

**Table:**

Bruker Kappa APEXII CCD area-detector diffractometer	6503 independent reflections
Radiation source: sealed tube	3926 reflections with *I* > 2σ(*I*)
graphite	*R*_int_ = 0.045
φ and ω scans	θ_max_ = 27.3°, θ_min_ = 2.3°
Absorption correction: multi-scan (*SADABS*; Bruker, 2007)	*h* = −11→11
*T*_min_ = 0.574, *T*_max_ = 0.755	*k* = −11→11
28784 measured reflections	*l* = −23→23

### Refinement

**Table:**

Refinement on *F*^2^	Primary atom site location: structure-invariant direct methods
Least-squares matrix: full	Secondary atom site location: difference Fourier map
*R*[*F*^2^ > 2σ(*F*^2^)] = 0.049	Hydrogen site location: inferred from neighbouring sites
*wR*(*F*^2^) = 0.125	H atoms treated by a mixture of independent and constrained refinement
*S* = 1.01	*w* = 1/[σ^2^(*F*_o_^2^) + (0.0517*P*)^2^ + 1.0386*P*] where *P* = (*F*_o_^2^ + 2*F*_c_^2^)/3
6503 reflections	(Δ/σ)_max_ < 0.001
394 parameters	Δρ_max_ = 0.79 e Å^−^^3^
3 restraints	Δρ_min_ = −0.78 e Å^−^^3^

### Special details

**Table:**

Geometry. Bond distances, angles *etc*. have been calculated using the rounded fractional coordinates. All su's are estimated from the variances of the (full) variance-covariance matrix. The cell e.s.d.'s are taken into account in the estimation of distances, angles and torsion angles
Refinement. Refinement on *F*^2^ for ALL reflections except those flagged by the user for potential systematic errors. Weighted *R*-factors *wR* and all goodnesses of fit *S* are based on *F*^2^, conventional *R*-factors *R* are based on *F*, with *F* set to zero for negative *F*^2^. The observed criterion of *F*^2^ > σ(*F*^2^) is used only for calculating -*R*-factor-obs *etc*. and is not relevant to the choice of reflections for refinement. *R*-factors based on *F*^2^ are statistically about twice as large as those based on *F*, and *R*-factors based on ALL data will be even larger.

### Fractional atomic coordinates and isotropic or equivalent isotropic displacement parameters (Å^2^)

**Table:**

	*x*	*y*	*z*	*U*_iso_*/*U*_eq_	
Br1	0.18494 (11)	0.21756 (9)	0.03270 (3)	0.1272 (4)	
Br2	0.67855 (10)	0.41062 (7)	0.04707 (4)	0.1172 (3)	
S1	0.30302 (9)	0.42033 (9)	0.38850 (5)	0.0285 (3)	
S2	0.65226 (10)	−0.10753 (12)	0.24430 (5)	0.0439 (3)	
Na1	0.52561 (13)	0.19278 (13)	0.50701 (7)	0.0316 (4)	
Na2	−0.05909 (14)	−0.09081 (14)	0.41846 (7)	0.0344 (4)	
O1	0.3932 (2)	0.3272 (3)	0.42249 (13)	0.0385 (8)	
O2	0.3634 (3)	0.5784 (2)	0.40534 (13)	0.0405 (8)	
O3	−0.0218 (2)	0.1828 (2)	0.46798 (12)	0.0319 (7)	
O4	−0.2643 (3)	0.1894 (3)	0.46144 (16)	0.0469 (9)	
O5	0.6421 (3)	−0.2446 (3)	0.19591 (15)	0.0580 (11)	
O6	0.7791 (3)	−0.0561 (3)	0.30905 (14)	0.0546 (10)	
O7	0.4069 (2)	−0.0348 (2)	0.39614 (12)	0.0332 (7)	
O8	0.1636 (2)	−0.0201 (3)	0.38076 (13)	0.0401 (8)	
O9	0.2820 (3)	0.1057 (2)	0.53176 (13)	0.0327 (7)	
N1	0.1423 (3)	0.3765 (3)	0.41057 (16)	0.0347 (9)	
N2	0.5022 (3)	−0.1155 (4)	0.27713 (17)	0.0491 (10)	
C1	0.2647 (4)	0.3679 (3)	0.28966 (19)	0.0328 (11)	
C2	0.3375 (5)	0.4607 (5)	0.2488 (2)	0.0538 (14)	
C3	0.3153 (6)	0.4152 (6)	0.1727 (3)	0.0702 (19)	
C4	0.2172 (6)	0.2803 (5)	0.1381 (2)	0.0633 (18)	
C5	0.1437 (7)	0.1906 (5)	0.1779 (3)	0.081 (2)	
C6	0.1672 (5)	0.2339 (4)	0.2550 (2)	0.0611 (14)	
C7	0.0134 (3)	0.4363 (3)	0.39122 (18)	0.0286 (10)	
C8	0.0127 (4)	0.5507 (4)	0.3489 (2)	0.0389 (11)	
C9	−0.1154 (4)	0.6063 (4)	0.3323 (2)	0.0446 (14)	
C10	−0.2422 (4)	0.5563 (4)	0.3587 (2)	0.0438 (14)	
C11	−0.2430 (4)	0.4413 (4)	0.3987 (2)	0.0367 (11)	
C12	−0.1186 (3)	0.3762 (3)	0.41430 (17)	0.0267 (10)	
C13	−0.1356 (4)	0.2409 (3)	0.45114 (18)	0.0291 (10)	
C14	0.6572 (4)	0.0334 (4)	0.1895 (2)	0.0457 (14)	
C15	0.6580 (6)	0.1769 (5)	0.2209 (2)	0.0680 (18)	
C16	0.6661 (6)	0.2892 (5)	0.1785 (3)	0.0773 (19)	
C17	0.6725 (6)	0.2564 (5)	0.1053 (3)	0.0692 (19)	
C18	0.6702 (6)	0.1148 (5)	0.0723 (3)	0.0756 (18)	
C19	0.6622 (5)	0.0020 (5)	0.1150 (2)	0.0612 (18)	
C20	0.3492 (4)	−0.1496 (4)	0.23536 (19)	0.0381 (11)	
C21	0.2373 (4)	−0.1206 (4)	0.27352 (18)	0.0322 (11)	
C22	0.0858 (4)	−0.1555 (4)	0.2323 (2)	0.0406 (12)	
C23	0.0440 (5)	−0.2195 (5)	0.1569 (2)	0.0541 (14)	
C24	0.1548 (5)	−0.2464 (5)	0.1205 (2)	0.0607 (16)	
C25	0.3066 (5)	−0.2097 (5)	0.1589 (2)	0.0558 (16)	
C26	0.2723 (4)	−0.0542 (3)	0.35624 (18)	0.0302 (11)	
H1	0.13370	0.30800	0.43760	0.0420*	
HW1	0.247 (8)	0.094 (7)	0.4844 (9)	0.1760*	
H2	0.51700	−0.09700	0.32540	0.0590*	
HW2	0.293 (8)	0.022 (4)	0.544 (3)	0.1760*	
H2A	0.40110	0.55330	0.27270	0.0640*	
H3	0.36620	0.47520	0.14470	0.0850*	
H5	0.07730	0.09980	0.15340	0.0980*	
H6	0.11740	0.17270	0.28280	0.0730*	
H8	0.09900	0.58880	0.33210	0.0470*	
H9	−0.11650	0.67970	0.30250	0.0530*	
H10	−0.32610	0.59930	0.34980	0.0520*	
H11	−0.32960	0.40600	0.41590	0.0440*	
H15	0.65300	0.19850	0.27100	0.0820*	
H16	0.66720	0.38630	0.19990	0.0930*	
H18	0.67400	0.09410	0.02200	0.0900*	
H19	0.66010	−0.09500	0.09310	0.0740*	
H22	0.01040	−0.13500	0.25630	0.0490*	
H23	−0.05850	−0.24410	0.13090	0.0650*	
H24	0.12740	−0.28970	0.06970	0.0730*	
H25	0.38120	−0.22540	0.13340	0.0670*	

### Atomic displacement parameters (Å^2^)

**Table:**

	*U*^11^	*U*^22^	*U*^33^	*U*^12^	*U*^13^	*U*^23^
Br1	0.2170 (9)	0.1298 (6)	0.0341 (3)	0.0553 (6)	0.0201 (4)	0.0057 (3)
Br2	0.2026 (8)	0.0792 (4)	0.0861 (5)	0.0425 (4)	0.0478 (5)	0.0360 (3)
S1	0.0220 (4)	0.0325 (4)	0.0320 (5)	0.0049 (3)	0.0073 (3)	0.0101 (3)
S2	0.0370 (5)	0.0752 (7)	0.0311 (5)	0.0319 (5)	0.0131 (4)	0.0143 (5)
Na1	0.0244 (7)	0.0336 (6)	0.0399 (8)	0.0075 (5)	0.0109 (6)	0.0117 (6)
Na2	0.0261 (7)	0.0479 (8)	0.0318 (7)	0.0108 (6)	0.0085 (6)	0.0104 (6)
O1	0.0250 (12)	0.0518 (14)	0.0440 (14)	0.0131 (11)	0.0083 (11)	0.0210 (12)
O2	0.0386 (14)	0.0347 (12)	0.0416 (15)	−0.0033 (11)	0.0075 (11)	0.0051 (11)
O3	0.0236 (12)	0.0375 (12)	0.0389 (13)	0.0098 (10)	0.0089 (10)	0.0160 (10)
O4	0.0295 (14)	0.0436 (14)	0.082 (2)	0.0143 (11)	0.0289 (13)	0.0282 (13)
O5	0.0666 (19)	0.0757 (19)	0.0493 (17)	0.0421 (16)	0.0262 (15)	0.0145 (14)
O6	0.0321 (14)	0.103 (2)	0.0388 (15)	0.0332 (14)	0.0083 (12)	0.0223 (15)
O7	0.0240 (12)	0.0496 (13)	0.0278 (12)	0.0119 (10)	0.0056 (10)	0.0092 (10)
O8	0.0240 (12)	0.0632 (15)	0.0354 (14)	0.0117 (11)	0.0105 (11)	0.0099 (12)
O9	0.0264 (12)	0.0397 (12)	0.0367 (13)	0.0106 (10)	0.0118 (11)	0.0126 (10)
N1	0.0278 (15)	0.0407 (15)	0.0464 (18)	0.0139 (12)	0.0161 (13)	0.0251 (14)
N2	0.0321 (17)	0.091 (2)	0.0281 (16)	0.0255 (17)	0.0076 (14)	0.0066 (16)
C1	0.0330 (19)	0.0325 (17)	0.0337 (19)	0.0104 (15)	0.0060 (16)	0.0079 (15)
C2	0.057 (3)	0.056 (2)	0.043 (2)	−0.002 (2)	0.013 (2)	0.012 (2)
C3	0.091 (4)	0.082 (3)	0.048 (3)	0.017 (3)	0.033 (3)	0.027 (3)
C4	0.100 (4)	0.063 (3)	0.031 (2)	0.032 (3)	0.011 (2)	0.011 (2)
C5	0.127 (5)	0.048 (3)	0.049 (3)	−0.001 (3)	0.001 (3)	0.003 (2)
C6	0.091 (3)	0.043 (2)	0.039 (2)	−0.002 (2)	0.011 (2)	0.0057 (19)
C7	0.0239 (17)	0.0301 (16)	0.0336 (18)	0.0083 (14)	0.0079 (14)	0.0076 (14)
C8	0.036 (2)	0.0375 (18)	0.050 (2)	0.0107 (16)	0.0171 (18)	0.0173 (17)
C9	0.049 (2)	0.0348 (19)	0.059 (3)	0.0169 (17)	0.016 (2)	0.0243 (18)
C10	0.033 (2)	0.041 (2)	0.063 (3)	0.0189 (16)	0.0107 (19)	0.0151 (18)
C11	0.0260 (18)	0.0369 (18)	0.051 (2)	0.0092 (15)	0.0145 (16)	0.0101 (16)
C12	0.0241 (17)	0.0291 (16)	0.0280 (17)	0.0074 (13)	0.0076 (14)	0.0048 (13)
C13	0.0289 (19)	0.0299 (16)	0.0317 (18)	0.0081 (14)	0.0126 (15)	0.0066 (14)
C14	0.041 (2)	0.067 (3)	0.033 (2)	0.0240 (19)	0.0076 (17)	0.0076 (18)
C15	0.096 (4)	0.077 (3)	0.033 (2)	0.035 (3)	0.014 (2)	−0.003 (2)
C16	0.112 (4)	0.060 (3)	0.057 (3)	0.027 (3)	0.016 (3)	−0.003 (2)
C17	0.097 (4)	0.066 (3)	0.051 (3)	0.024 (3)	0.024 (3)	0.016 (2)
C18	0.124 (4)	0.074 (3)	0.037 (2)	0.035 (3)	0.025 (3)	0.012 (2)
C19	0.095 (4)	0.065 (3)	0.034 (2)	0.031 (2)	0.025 (2)	0.012 (2)
C20	0.0310 (19)	0.057 (2)	0.0279 (19)	0.0157 (17)	0.0054 (16)	0.0082 (16)
C21	0.0297 (19)	0.0387 (18)	0.0288 (18)	0.0075 (15)	0.0068 (15)	0.0098 (15)
C22	0.031 (2)	0.057 (2)	0.031 (2)	0.0047 (17)	0.0067 (16)	0.0079 (17)
C23	0.035 (2)	0.079 (3)	0.041 (2)	0.009 (2)	−0.0014 (19)	0.008 (2)
C24	0.053 (3)	0.084 (3)	0.031 (2)	0.011 (2)	−0.005 (2)	−0.007 (2)
C25	0.050 (3)	0.084 (3)	0.036 (2)	0.025 (2)	0.012 (2)	0.003 (2)
C26	0.0288 (19)	0.0336 (17)	0.0322 (19)	0.0081 (14)	0.0100 (16)	0.0143 (14)

### Geometric parameters (Å, °)

**Table:**

Br1—C4	1.894 (4)	C7—C8	1.398 (5)
Br2—C17	1.898 (5)	C7—C12	1.401 (4)
S1—O1	1.428 (3)	C8—C9	1.369 (5)
S1—O2	1.427 (2)	C9—C10	1.372 (5)
S1—N1	1.598 (3)	C10—C11	1.375 (5)
S1—C1	1.762 (3)	C11—C12	1.391 (5)
S2—O5	1.426 (3)	C12—C13	1.501 (4)
S2—O6	1.435 (3)	C14—C19	1.376 (5)
S2—N2	1.605 (3)	C14—C15	1.376 (6)
S2—C14	1.754 (4)	C15—C16	1.382 (6)
Na1—O1	2.408 (3)	C16—C17	1.358 (8)
Na1—O7	2.613 (2)	C17—C18	1.365 (7)
Na1—O9	2.377 (3)	C18—C19	1.390 (6)
Na1—O4^i^	2.257 (3)	C20—C21	1.405 (5)
Na1—O7^ii^	2.514 (2)	C20—C25	1.387 (5)
Na1—O2^iii^	2.386 (2)	C21—C22	1.390 (5)
Na2—O3	2.508 (2)	C21—C26	1.507 (5)
Na2—O8	2.292 (2)	C22—C23	1.378 (5)
Na2—O6^iv^	2.346 (3)	C23—C24	1.371 (6)
Na2—O3^v^	2.397 (2)	C24—C25	1.377 (6)
Na2—O9^v^	2.393 (3)	C2—H2A	0.9300
O3—C13	1.269 (4)	C3—H3	0.9300
O4—C13	1.244 (5)	C5—H5	0.9300
O7—C26	1.265 (4)	C6—H6	0.9300
O8—C26	1.253 (4)	C8—H8	0.9300
O9—HW2	0.86 (4)	C9—H9	0.9300
O9—HW1	0.849 (19)	C10—H10	0.9300
N1—C7	1.404 (4)	C11—H11	0.9300
N2—C20	1.405 (5)	C15—H15	0.9300
N1—H1	0.8600	C16—H16	0.9300
N2—H2	0.8600	C18—H18	0.9300
C1—C6	1.364 (5)	C19—H19	0.9300
C1—C2	1.380 (5)	C22—H22	0.9300
C2—C3	1.369 (6)	C23—H23	0.9300
C3—C4	1.372 (7)	C24—H24	0.9300
C4—C5	1.352 (7)	C25—H25	0.9300
C5—C6	1.382 (6)		
			
Br1···C23^vi^	3.691 (4)	C22···C6	3.488 (5)
Br1···H19^vii^	3.1600	C22···Na2	3.939 (4)
Br1···H23^vi^	3.0600	C22···C5	3.462 (6)
S1···H8	2.8000	C22···C9^viii^	3.541 (5)
S2···H25	2.7900	C23···Br1^vi^	3.691 (4)
Na1···C11^i^	3.849 (4)	C25···C14	3.430 (6)
Na2···C8^viii^	3.633 (4)	C25···O5	3.103 (5)
Na2···C9^viii^	2.926 (4)	C25···C3^viii^	3.537 (7)
Na2···C10^viii^	3.290 (4)	C1···H8	2.9000
Na2···C22	3.939 (4)	C9···H16^iv^	3.0200
Na1···H2^ii^	3.4000	C9···H22^x^	3.0800
Na1···HW2^ii^	3.01 (6)	C10···H16^iv^	3.0000
Na1···H11^i^	3.0400	C11···H15^iv^	2.8800
Na2···HW1	2.90 (7)	C12···H15^iv^	3.0400
Na2···HW2	3.44 (7)	C13···HW2^v^	2.60 (4)
Na2···H9^viii^	2.6900	C13···H1	2.5000
Na2···H10^viii^	3.2900	C14···H25	3.0100
Na2···H22	3.1700	C19···H25	3.1000
Na2···H1^v^	3.6300	C20···H2A^viii^	3.0300
O1···O9	3.229 (3)	C22···H6	3.0000
O2···C8	3.117 (5)	C22···H9^viii^	2.8000
O3···N1	2.588 (3)	C22···H5	2.9400
O3···O9^v^	3.212 (3)	C23···H5	2.9300
O3···O9	3.054 (3)	C25···H3^viii^	3.0600
O4···O7^iv^	3.195 (3)	C26···HW1	2.65 (4)
O4···O9^v^	2.730 (3)	C26···H2	2.5200
O5···C25	3.103 (5)	C26···H6	3.0700
O6···O9^ii^	3.171 (3)	H1···O3	1.8800
O6···C13^i^	3.378 (4)	H1···C13	2.5000
O7···O9	3.184 (3)	H1···HW1	2.5800
O7···O4^i^	3.195 (3)	H1···Na2^v^	3.6300
O7···O9^ii^	3.124 (3)	HW1···Na2	2.90 (7)
O7···N2	2.595 (4)	HW1···O8	1.97 (3)
O8···O9	2.768 (3)	HW1···C26	2.65 (4)
O9···C13^v^	3.311 (3)	HW1···H1	2.5800
O9···O8	2.768 (3)	H2···O7	1.9000
O9···O3	3.054 (3)	H2···C26	2.5200
O9···O3^v^	3.212 (3)	H2···Na1^ii^	3.4000
O9···Na1^ii^	3.634 (3)	H2···O9^ii^	2.9100
O9···O4^v^	2.730 (3)	H2···HW2^ii^	2.5800
O9···O6^ii^	3.171 (3)	HW2···Na2	3.44 (7)
O9···O1	3.229 (3)	HW2···Na1^ii^	3.01 (6)
O9···O7	3.184 (3)	HW2···O4^v^	1.91 (4)
O9···Na2	3.390 (3)	HW2···C13^v^	2.60 (5)
O9···C26	3.347 (4)	HW2···H2^ii^	2.5800
O9···O7^ii^	3.124 (3)	H2A···O2	2.5200
O1···H11^i^	2.5300	H2A···C20^x^	3.0300
O1···HW1	2.81 (6)	H2A···H10^i^	2.5300
O2···H2A	2.5200	H3···C25^x^	3.0600
O2···H8	2.5500	H5···C22	2.9400
O3···HW1	2.72 (7)	H5···C23	2.9300
O3···HW2^v^	2.76 (6)	H6···O8	2.7400
O3···H1	1.8800	H6···N1	2.7500
O4···HW2^v^	1.91 (4)	H6···C22	3.0000
O4···H11	2.4100	H6···C26	3.0700
O5···H25	2.4800	H8···S1	2.8000
O5···H9^ix^	2.9100	H8···O2	2.5500
O5···H19	2.5100	H8···C1	2.9000
O6···H9^ix^	2.8000	H9···Na2^x^	2.6900
O6···H22^i^	2.6900	H9···O5^xi^	2.9100
O6···HW2^ii^	2.91 (6)	H9···O6^xi^	2.8000
O7···H2	1.9000	H9···C22^x^	2.8000
O7···HW1	2.72 (6)	H9···H22^x^	2.2400
O7···HW2^ii^	2.72 (7)	H10···Na2^x^	3.2900
O8···H6	2.7400	H10···H2A^iv^	2.5300
O8···H22	2.4100	H11···Na1^iv^	3.0400
O8···HW1	1.97 (3)	H11···O1^iv^	2.5300
O9···H2^ii^	2.9100	H11···O4	2.4100
N1···O3	2.588 (3)	H15···C11^i^	2.8800
N2···O7	2.595 (4)	H15···C12^i^	3.0400
N1···H6	2.7500	H16···C9^i^	3.0200
C1···C8	3.391 (5)	H16···C10^i^	3.0000
C3···C25^x^	3.537 (7)	H19···O5	2.5100
C5···C22	3.462 (6)	H19···Br1^vii^	3.1600
C6···C21	3.519 (5)	H22···Na2	3.1700
C6···C22	3.488 (5)	H22···O6^iv^	2.6900
C8···C1	3.391 (5)	H22···O8	2.4100
C8···Na2^x^	3.633 (4)	H22···C9^viii^	3.0800
C8···O2	3.117 (5)	H22···H9^viii^	2.2400
C9···Na2^x^	2.926 (4)	H23···Br1^vi^	3.0600
C9···C22^x^	3.541 (5)	H25···S2	2.7900
C10···Na2^x^	3.290 (4)	H25···O5	2.4800
C11···Na1^iv^	3.849 (4)	H25···C14	3.0100
C14···C25	3.430 (6)	H25···C19	3.1000
C21···C6	3.519 (5)		
			
O1—S1—O2	118.46 (16)	C1—C6—C5	119.0 (4)
O1—S1—N1	105.18 (14)	N1—C7—C12	118.2 (3)
O1—S1—C1	108.71 (15)	N1—C7—C8	122.1 (3)
O2—S1—N1	111.26 (16)	C8—C7—C12	119.8 (3)
O2—S1—C1	106.36 (14)	C7—C8—C9	119.8 (3)
N1—S1—C1	106.28 (16)	C8—C9—C10	121.5 (3)
O5—S2—O6	118.57 (17)	C9—C10—C11	118.7 (4)
O5—S2—N2	110.77 (18)	C10—C11—C12	122.1 (3)
O5—S2—C14	107.61 (17)	C7—C12—C11	117.9 (3)
O6—S2—N2	105.12 (16)	C7—C12—C13	123.5 (3)
O6—S2—C14	107.31 (17)	C11—C12—C13	118.4 (3)
N2—S2—C14	106.88 (18)	O3—C13—O4	123.6 (3)
O1—Na1—O7	83.84 (8)	O3—C13—C12	119.3 (3)
O1—Na1—O9	84.90 (8)	O4—C13—C12	117.0 (3)
O1—Na1—O4^i^	100.77 (10)	C15—C14—C19	119.4 (4)
O1—Na1—O7^ii^	164.17 (9)	S2—C14—C15	120.3 (3)
O1—Na1—O2^iii^	89.44 (9)	S2—C14—C19	120.4 (3)
O7—Na1—O9	79.17 (8)	C14—C15—C16	120.5 (4)
O4^i^—Na1—O7	81.64 (9)	C15—C16—C17	119.3 (4)
O7—Na1—O7^ii^	94.55 (7)	C16—C17—C18	121.6 (5)
O2^iii^—Na1—O7	171.85 (9)	Br2—C17—C16	118.9 (4)
O4^i^—Na1—O9	159.29 (10)	Br2—C17—C18	119.5 (4)
O7^ii^—Na1—O9	79.34 (8)	C17—C18—C19	119.0 (5)
O2^iii^—Na1—O9	104.87 (10)	C14—C19—C18	120.2 (4)
O4^i^—Na1—O7^ii^	94.55 (9)	N2—C20—C21	117.8 (3)
O2^iii^—Na1—O4^i^	95.16 (11)	N2—C20—C25	122.4 (4)
O2^iii^—Na1—O7^ii^	93.16 (8)	C21—C20—C25	119.8 (4)
O3—Na2—O8	85.24 (9)	C20—C21—C22	117.8 (3)
O3—Na2—O6^iv^	87.43 (9)	C20—C21—C26	123.8 (3)
O3—Na2—O3^v^	101.63 (8)	C22—C21—C26	118.4 (3)
O3—Na2—O9^v^	81.86 (8)	C21—C22—C23	122.0 (4)
O6^iv^—Na2—O8	95.51 (10)	C22—C23—C24	119.4 (4)
O3^v^—Na2—O8	103.49 (9)	C23—C24—C25	120.3 (3)
O8—Na2—O9^v^	167.10 (10)	C20—C25—C24	120.6 (4)
O3^v^—Na2—O6^iv^	159.49 (10)	O8—C26—C21	116.6 (3)
O6^iv^—Na2—O9^v^	84.00 (10)	O7—C26—O8	124.2 (3)
O3^v^—Na2—O9^v^	79.20 (8)	O7—C26—C21	119.3 (3)
S1—O1—Na1	165.86 (15)	C1—C2—H2A	120.00
S1—O2—Na1^iii^	151.44 (15)	C3—C2—H2A	120.00
Na2—O3—C13	117.57 (18)	C2—C3—H3	120.00
Na2—O3—Na2^v^	78.37 (7)	C4—C3—H3	120.00
Na2^v^—O3—C13	127.0 (2)	C4—C5—H5	120.00
Na1^iv^—O4—C13	156.3 (2)	C6—C5—H5	120.00
S2—O6—Na2^i^	152.55 (18)	C1—C6—H6	121.00
Na1—O7—C26	121.54 (18)	C5—C6—H6	121.00
Na1—O7—Na1^ii^	85.45 (8)	C7—C8—H8	120.00
Na1^ii^—O7—C26	122.43 (18)	C9—C8—H8	120.00
Na2—O8—C26	149.8 (2)	C8—C9—H9	119.00
Na1—O9—Na2^v^	162.49 (11)	C10—C9—H9	119.00
Na1—O9—HW2	100 (5)	C9—C10—H10	121.00
HW1—O9—HW2	110 (6)	C11—C10—H10	121.00
Na1—O9—HW1	86 (5)	C10—C11—H11	119.00
Na2^v^—O9—HW2	91 (5)	C12—C11—H11	119.00
Na2^v^—O9—HW1	104 (5)	C14—C15—H15	120.00
S1—N1—C7	127.9 (2)	C16—C15—H15	120.00
S2—N2—C20	126.9 (3)	C15—C16—H16	120.00
S1—N1—H1	116.00	C17—C16—H16	120.00
C7—N1—H1	116.00	C17—C18—H18	120.00
S2—N2—H2	117.00	C19—C18—H18	120.00
C20—N2—H2	117.00	C14—C19—H19	120.00
S1—C1—C6	119.4 (3)	C18—C19—H19	120.00
C2—C1—C6	121.0 (3)	C21—C22—H22	119.00
S1—C1—C2	119.5 (3)	C23—C22—H22	119.00
C1—C2—C3	119.4 (4)	C22—C23—H23	120.00
C2—C3—C4	119.4 (5)	C24—C23—H23	120.00
Br1—C4—C3	119.5 (4)	C23—C24—H24	120.00
Br1—C4—C5	119.2 (4)	C25—C24—H24	120.00
C3—C4—C5	121.3 (4)	C20—C25—H25	120.00
C4—C5—C6	120.0 (4)	C24—C25—H25	120.00
			
O1—S1—O2—Na1^iii^	−52.3 (4)	Na2^v^—O3—C13—C12	138.1 (2)
N1—S1—O2—Na1^iii^	69.8 (4)	Na2^v^—O3—C13—O4	−44.1 (4)
C1—S1—O2—Na1^iii^	−174.9 (3)	Na2—O3—C13—C12	−125.7 (2)
O1—S1—N1—C7	179.8 (3)	Na2—O3—C13—O4	52.2 (4)
O2—S1—N1—C7	50.3 (3)	Na1^iv^—O4—C13—C12	−59.0 (7)
C1—S1—N1—C7	−65.1 (3)	Na1^iv^—O4—C13—O3	123.2 (5)
O2—S1—C1—C6	−161.3 (3)	Na1^ii^—O7—C26—C21	−108.4 (3)
N1—S1—C1—C6	−42.6 (4)	Na1—O7—C26—O8	−33.8 (4)
N1—S1—C1—C2	139.5 (3)	Na1^ii^—O7—C26—O8	72.4 (3)
O1—S1—C1—C2	−107.8 (3)	Na1—O7—C26—C21	145.4 (2)
O2—S1—C1—C2	20.8 (4)	Na2—O8—C26—C21	76.0 (5)
O1—S1—C1—C6	70.2 (4)	Na2—O8—C26—O7	−104.8 (4)
C14—S2—N2—C20	61.3 (4)	S1—N1—C7—C12	177.4 (2)
O5—S2—N2—C20	−55.7 (4)	S1—N1—C7—C8	−1.4 (5)
O6—S2—N2—C20	175.1 (3)	S2—N2—C20—C21	−167.0 (3)
O5—S2—C14—C19	−4.6 (4)	S2—N2—C20—C25	12.9 (6)
O5—S2—C14—C15	176.2 (4)	C6—C1—C2—C3	−2.2 (7)
O6—S2—C14—C15	−55.1 (4)	S1—C1—C2—C3	175.7 (4)
C14—S2—O6—Na2^i^	−171.6 (4)	S1—C1—C6—C5	−176.9 (4)
O6—S2—C14—C19	124.1 (3)	C2—C1—C6—C5	1.0 (7)
N2—S2—C14—C19	−123.6 (4)	C1—C2—C3—C4	2.1 (8)
N2—S2—O6—Na2^i^	74.9 (4)	C2—C3—C4—Br1	179.5 (4)
O5—S2—O6—Na2^i^	−49.6 (4)	C2—C3—C4—C5	−0.9 (8)
N2—S2—C14—C15	57.2 (4)	Br1—C4—C5—C6	179.3 (4)
O9^ii^—Na1^ii^—O7—C26	157.1 (2)	C3—C4—C5—C6	−0.3 (9)
O7^ii^—Na1^ii^—O7—Na1	0.02 (12)	C4—C5—C6—C1	0.2 (8)
O7^ii^—Na1—O7—Na1^ii^	−0.02 (11)	C12—C7—C8—C9	2.0 (5)
O4^i^—Na1—O7—Na1^ii^	93.94 (9)	N1—C7—C12—C11	176.5 (3)
O9—Na1—O7—Na1^ii^	−78.22 (8)	N1—C7—C8—C9	−179.2 (3)
O7^ii^—Na1—O7—C26	125.6 (2)	C8—C7—C12—C11	−4.7 (5)
O4^v^—Na1^ii^—O7—Na1	81.95 (9)	C8—C7—C12—C13	172.0 (3)
O7^iv^—Na1^iv^—O4—C13	159.6 (6)	N1—C7—C12—C13	−6.9 (5)
O9^iv^—Na1^iv^—O4—C13	−178.2 (5)	C7—C8—C9—C10	2.5 (5)
O1—Na1—O7—Na1^ii^	−164.18 (8)	C8—C9—C10—C11	−4.1 (5)
O1^iii^—Na1^iii^—O2—S1	33.6 (4)	C9—C10—C11—C12	1.2 (5)
O9^iii^—Na1^iii^—O2—S1	118.2 (4)	C10—C11—C12—C7	3.2 (5)
O7^ii^—Na1^ii^—O7—C26	−124.8 (2)	C10—C11—C12—C13	−173.7 (3)
O4^i^—Na1—O7—C26	−140.4 (2)	C11—C12—C13—O3	−177.8 (3)
O1^iv^—Na1^iv^—O4—C13	77.6 (6)	C7—C12—C13—O4	−172.4 (3)
O4^v^—Na1^ii^—O7—C26	−42.9 (2)	C11—C12—C13—O4	4.2 (4)
O7^v^—Na1^iv^—O4—C13	−106.5 (6)	C7—C12—C13—O3	5.6 (5)
O9^ii^—Na1^ii^—O7—Na1	−78.07 (7)	S2—C14—C19—C18	−178.3 (4)
O9—Na1—O7—C26	47.4 (2)	S2—C14—C15—C16	178.2 (4)
O1—Na1—O7—C26	−38.6 (2)	C15—C14—C19—C18	0.9 (7)
O9—Na2^v^—O3—C13	−164.9 (2)	C19—C14—C15—C16	−1.0 (7)
O3^ii^—Na2^i^—O6—S2	−34.1 (6)	C14—C15—C16—C17	0.3 (8)
O6^ii^—Na2^v^—O3—Na2	114.8 (3)	C15—C16—C17—Br2	178.5 (4)
O6^iv^—Na2—O8—C26	−104.8 (4)	C15—C16—C17—C18	0.4 (9)
O3^v^—Na2—O8—C26	67.4 (4)	C16—C17—C18—C19	−0.5 (9)
O3—Na2—O8—C26	168.3 (4)	Br2—C17—C18—C19	−178.5 (4)
O8^v^—Na2^v^—O3—Na2	−87.84 (9)	C17—C18—C19—C14	−0.2 (8)
O9—Na2^v^—O3—Na2	79.25 (7)	C21—C20—C25—C24	−2.4 (6)
O3^v^—Na2^v^—O3—Na2	0.00 (8)	C25—C20—C21—C22	0.6 (6)
O3^v^—Na2—O3—C13	−125.8 (2)	N2—C20—C21—C22	−179.5 (3)
O9^v^—Na2—O3—C13	−48.7 (2)	N2—C20—C21—C26	0.5 (5)
O8—Na2—O3—Na2^v^	−102.82 (8)	C25—C20—C21—C26	−179.4 (3)
O6^iv^—Na2—O3—Na2^v^	161.43 (9)	N2—C20—C25—C24	177.7 (4)
O3^v^—Na2—O3—Na2^v^	0.00 (8)	C26—C21—C22—C23	−178.5 (4)
O9^v^—Na2—O3—Na2^v^	77.13 (8)	C20—C21—C22—C23	1.4 (6)
O3^i^—Na2^i^—O6—S2	−151.3 (4)	C22—C21—C26—O7	173.4 (3)
O8^i^—Na2^i^—O6—S2	123.8 (4)	C20—C21—C26—O7	−6.5 (5)
O6^iv^—Na2—O3—C13	35.6 (2)	C20—C21—C26—O8	172.7 (3)
O8^v^—Na2^v^—O3—C13	28.1 (2)	C22—C21—C26—O8	−7.3 (5)
O9^ii^—Na2^i^—O6—S2	−69.2 (4)	C21—C22—C23—C24	−1.7 (6)
O3^v^—Na2^v^—O3—C13	115.9 (2)	C22—C23—C24—C25	−0.1 (7)
O6^ii^—Na2^v^—O3—C13	−129.3 (3)	C23—C24—C25—C20	2.1 (7)
O8—Na2—O3—C13	131.3 (2)		

Symmetry codes: (i) *x*+1, *y*, *z*; (ii) −*x*+1, −*y*, −*z*+1; (iii) −*x*+1, −*y*+1, −*z*+1; (iv) *x*−1, *y*, *z*; (v) −*x*, −*y*, −*z*+1; (vi) −*x*, −*y*, −*z*; (vii) −*x*+1, −*y*, −*z*; (viii) *x*, *y*−1, *z*; (ix) *x*+1, *y*−1, *z*; (x) *x*, *y*+1, *z*; (xi) *x*−1, *y*+1, *z*.

### Hydrogen-bond geometry (Å, °)

**Table:**

*D*—H···*A*	*D*—H	H···*A*	*D*···*A*	*D*—H···*A*
N1—H1···O3	0.86	1.88	2.588 (3)	138
O9—HW1···O8	0.849 (19)	1.97 (3)	2.768 (3)	156 (6)
N2—H2···O7	0.86	1.90	2.595 (4)	137
O9—HW2···O4^v^	0.86 (4)	1.91 (4)	2.730 (3)	160 (6)
C2—H2A···O2	0.93	2.52	2.886 (4)	104
C8—H8···O2	0.93	2.55	3.117 (5)	120
C11—H11···O1^iv^	0.93	2.53	3.429 (4)	162
C11—H11···O4	0.93	2.41	2.732 (5)	100
C19—H19···O5	0.93	2.51	2.889 (5)	105
C22—H22···O8	0.93	2.41	2.736 (4)	101
C25—H25···O5	0.93	2.48	3.103 (5)	124

Symmetry codes: (v) −*x*, −*y*, −*z*+1; (iv) *x*−1, *y*, *z*.
